# Rapid Emergence and Evolution of SARS-CoV-2 Intrahost Variants among COVID-19 Patients with Prolonged Infections, Singapore 

**DOI:** 10.3201/eid3108.241419

**Published:** 2025-08

**Authors:** Yvonne C.F. Su, Michael A. Zeller, Peter Cronin, Rong Zhang, Yan Zhuang, Jordan Ma, Foong Ying Wong, Giselle G.K. Ng, Áine O’Toole, Andrew Rambaut, Jenny G. Low, Gavin J.D. Smith

**Affiliations:** Programme in Emerging Infectious Diseases, Duke-NUS Medical School, Singapore (Y.C.F. Su, M.A. Zeller, P. Cronin, R. Zhang, Y. Zhang, J. Ma, F.Y. Wong, G.G.K. Ng, J.G. Low, G.J.D. Smith); Institute of Evolutionary Biology, University of Edinburgh, Edinburgh, Scotland, UK (A. O’Toole, A. Rambaut); Singapore General Hospital, Singapore (J.G. Low)

**Keywords:** COVID-19, respiratory infections, severe acute respiratory syndrome coronavirus 2, SARS-CoV-2, SARS, coronavirus disease, zoonoses, viruses, coronavirus virus evolution, intrahost, genetic diversity, variants, Singapore

## Abstract

The evolution and spread of SARS-CoV-2 variants have driven successive waves of global COVID-19 outbreaks, yet the longitudinal dynamics of intrahost variation within the same patient remain less clear. We conducted a longitudinal cohort study by deep sequencing 198 swab samples collected from COVID-19 patients with varying infection durations. Our analysis showed that prolonged infections enhanced viral genomic diversity, leading to emergence of co-occurring variants that maintained high (>20%) frequency and became dominant in virus populations. We observed heterogeneous intrahost dynamics among individual patients, 2 of whom exhibited a minor variant of the spike D614G substitution over the course of infection. The increase in intrahost variants strongly correlated with prolonged infections, highlighting the complex interplay between viral diversity and host factors. This study revealed the intricate evolutionary mechanisms driving the emergence of de novo variants and lineage dominance, which could inform development of effective vaccine candidates and strategies to protect public health.

The COVID-19 pandemic, caused by the zoonotic SARS-CoV-2 virus, led to an unprecedented global crisis in the 21st Century. The application of advanced sequencing technologies enabled rapid identification of emerging de novo SARS-CoV-2 variants and helped elucidate how prevailing lineages were arising and spreading. Singapore was among the first countries outside China to implement rigorous COVID-19 surveillance. During the early period of the SARS-CoV-2 outbreak, from late January to early March 2020, viruses from multiple patients in Singapore exhibited a long, 382-nt deletion mutation in the open reading frame (ORF) regions ORF7b and ORF8 (*1*) that was later eliminated in the population, possibly because of the reduction in case counts resulting from the country’s effective control measures (*2*). ORF8 deletions of varying lengths have repeatedly reemerged in subsequent major variants, including Alpha, Delta, and Omicron XBB.1 (*3**–**6*).

Studies investigating the intrahost dynamics of SARS-CoV-2 virus have demonstrated that intrahost single-nucleotide variants (iSNVs) are associated with virus shredding (*7*), transmission bottlenecks (*8*,*9*), purifying selection (*10*), immunosuppression (*11*), and vaccinations (*12*). Growing attention has been directed toward determining the complexity of viral evolution during persistent infections within hosts (*13*–*15*; M. Ghafari et al., unpub. data, https://doi.org/10.1101/2024.06.21.24309297; N. Rutsinsky et al., unpub. data, https://doi.org/10.1101/2024.11.23.624482). However, the intrahost evolutionary dynamics of SARS-CoV-2 in Singapore remain largely uncharacterized. We investigated the longitudinal intrahost variation of SARS-CoV-2 in patients with varying durations of infection during early 2020. 

## Materials and Methods

### Sample Collection

During March–May 2020, we collected a total of 198 nasopharyngeal swab samples from 20 adult hospitalized COVID-19 patients at Singapore General Hospital (SGH). Epidemiologic and clinical data included age, sex, height, weight, body mass index, underlying conditions, intensive care unit (ICU) admission, infection duration, leukocyte count, C-reactive protein (CRP) count, and remdesivir treatment.

### RNA Extraction and Next-Generation Sequencing

We extracted viral RNA from swab samples and tested for the SARS-CoV-2 RNA-dependent RNA polymerase gene, as previously described (*16*). We generated complete SARS-CoV-2 genomes via next-generation sequencing. We conducted library preparation by using the Illumina RNA Prep Enrichment Kit (https://www.illumina.com) and performed viral enrichment by using Respiratory Virus Oligo Panel (Illumina), following manufacturer protocols. We quantified libraries by using the Qubit dsDNA HS Assay Kit (Thermo Fisher Scientific, https://www.thermofisher.com) and quality-checked by using 2100 Bioanalyzer (Agilent Technologies, https://www.agilent.com). We ran pooled libraries on an Illumina MiSeq platform at 2 × 250 bp. We used Trimmomatic version 0.39 (*17*) to quality-trim reads using a minimum read quality of 20, leading/trailing quality of 10, and a minimum length of 50. For samples collected on the first day of swab sampling, we mapped trimmed paired reads to the wild-type SARS-CoV-2 reference genome (GenBank accession no. NC_045512.2) using Burrow-Wheeler Aligner–Maximal Exact Match (*18*) with UGENE version 42 (*19*). We used Pangolin version 4.3.1 (*20*) to assign Pango lineages to SARS-CoV-2 genomes from patients (GISAID accession nos. EPI_ISL_19591944–57).

### iSNV Analyses

To investigate within-host evolutionary dynamics of SARS-CoV-2, we used daily nasopharyngeal swab specimens collected from the 20 participants hospitalized at SGH over the course of infection, spanning up to 40 days. We deep sequenced all 198 samples, yielding 92 complete genomes from serial timepoints (Table 1). We used SAMtools (*21*) to identified iSNVs and generate mpileup files, then performed variant calling by using VarScan version 2.3.4 (*22*). 

**Table 1:** Epidemiologic and clinical characteristics of hospitalized patients in a study of rapid emergence and evolution of SARS-CoV-2 intrahost variants among COVID-19 patients with prolonged infections, Singapore*

ID	Age,y/sex	BMI	Underlying conditions†	ICU admission	No. days hospitalized	Remdesivir treatment	Median lymphocyte count‡	Median CRP, mg/L	Median leukocyte count‡	Long-term medication	Pangolin lineage
P1	29/F	23.3	N	N	5	N	0.71	0	3.95	N	B.6.6
P2	48/M	26.8	N	N	13	Y	2.28	18.5	5.93	N	B.6
P3	70/M	22.5	Y	Y	40	Y	0.79	236.5	10.89	Y	B.6.6
P4	65/M	NA	Y	N	30	N	1.22	51.2	6.89	Y	B.6.6
P5	67/F	30.9	N	N	14	Y	1.05	122	4.61	N	B.1.104
P6	28/M	NA	N	N	7	N	1.75	0	4.28	N	B.6.3
P7	64/M	31.5	Y	N	16	N	2.32	12.6	5.09	Y	B.6.6
P8	29/M	20.8	N	N	5	N	1.14	0	4.45	N	B.6.6
PP9	35/F	21.6	N	N	7	N	1.44	0.9	4.66	N	B.1.1
P10	25/M	21.7	N	N	11	N	1.45	0	3.80	N	B.6.6
P11	32/M	27.3	N	N	4	N	0.87	0	7.00	N	B.1.1
P12	41/M	NA	N	N	6	N	0.98	0	4.99	N	B.6.6
P13	37/M	28.7	N	N	6	N	0.92	0	2.48	N	B.6.6
P14	34/F	NA	N	N	5	N	1.82	0	5.29	N	B.6.6
P15	54/M	NA	N	N	12	N	1.18	0.3	8.83	N	B.6.6
P16	21/F	NA	N	N	8	N	1.31	31.9	4.98	N	B.1.1
P17	50/M	31.8	N	N	3	Y	1.61	73	4.65	N	B.6
P18	37/M	NA	N	N	5	N	3.31	0	6.89	N	ND
P19	39/M	14.7	N	N	5	N	0.74	0	3.73	N	B.1.1
P20	61/F	25.8	Y	Y	30	Y	1.47	158	9.06	Y	B.6

*BMI, body mass index; CRP, C-reactive protein; ICU, intensive care unit; ID, patient identification; ND, not determined; P, patient. †Including hypertension or hyperlipidemia. ‡Value × 10^9^ cells/L.

We applied rigorous quality control steps to reduce sequencing errors. First, we trimmed and filtered reads with a minimum Phred score >30. We required variants to have sequencing depth of 200–60,000 reads, a p value of <0.01, variant read depth >10×, and genome coverage >95%. Then we used the strand-filter parameter to remove variants detected predominantly on either the forward or reverse strand but not both. To minimize false-positive results and exclude potentially fixed variants, we only retained variants with frequencies of 5%–95%, following widely used minor allele frequency cutoffs (*13*,*23*,*24*). That threshold is well above the reported error rates for next-generation sequencing platforms, ensuring reliable variant detection (*25*). For samples collected on the first day of hospitalization, we used SnpEff (*26*) to perform variant annotation on the basis of the wild-type reference genome (*7*,*8*,*27*,*28*). For longitudinal samples, we based annotations on the reference genome of the first confirmed Singapore case (BetaCoV/Singapore/2/2020; GISAID accession no. EPI_ISL_406973) that differs from the wild-type reference genome by a single nucleotide. We used MAFFT (https://mafft.cbrc.jp) to conducted genome alignments in Geneious Prime version 2022.1.1 (https://www.geneious.com), then manually refined.

We identified iSNVs representing subconsensus genetic diversity on the basis of nucleotide composition at each genomic position (*27*,*29*) (Appendix 1 Table 1). We found iSNV counts and frequencies were consistent when we used either the wild-type or BetaCoV/Singapore/2/2020 reference genomes. We visualized iSNV frequencies and distributions by using the ggplot2 package (https://github.com/tidyverse/ggplot2) and custom scripts in R (The R Project for Statistical Computing, https://www.r-project.org). We used the ComplexHeatmap package (*30*) in R to display high (>20%) frequency iSNVs as heatmaps. To assess variation of iSNV counts and frequencies over the course of infection, we stratified patients by illness duration into acute (<7 days) and prolonged (>8 days) groups. That cutoff reflects earlier studies indicating that mild or moderate COVID-19 cases typically resolve within a week, but severe cases exhibit extended viral shedding (*31*–*34*). For each patient, we quantified the number of synonymous, nonsynonymous, and nonsense (stop) variants. We normalized iSNV counts per gene by length (kb). We visualized normalized values across all sampling days per patient as bar plots, indicating relative proportions of synonymous and nonsynonymous variants.

### Correlation and Linear Regression Analyses

We used the corrplot package version 0.92 in R (https://CRAN.R-project.org/package=corrplot) to calculate Pearson correlation coefficients (*r*) for assessing associations between iSNV counts and 11 clinical variables and considered p<0.05 statistically significant. We defined iSNV counts as the number of unique genomic positions with a variant detected in >1 sample per patient. We classified correlation strength as very strong (*r*>0.7), strong (*r* = 0.5–0.7), moderate (*r* = 0.3–0.5), or weak (*r*<0.3). We further tested associations between iSNV counts and clinical parameters by using a negative binomial regression model with a log-link function in the MASS package (*35*) in R. We performed Wilcoxon tests to compare factors between 2 groups. We used the Benjamini-Hochberg method to correct all p values for false discovery rate.

### Ethics Considerations

This study was approved by the SingHealth Centralized Institutional Review Board (CIRB reference no. 2018/3045) and the National University of Singapore (NUS) Institutional Review Board (NUS-IRB reference code 2022-320). Written informed consent was obtained from all participants. All recruited COVID-19 patients were hospitalized during the early phase of the pandemic, isolated in negative pressure rooms, and discharged only after 2 consecutive negative quantitative PCR (qPCR) tests. All samples were de-identified and processed under Biosafety Level 3 conditions.

## Results

### Clinical Characteristics of Hospitalized COVID-19 Patients

**Figure 1:**
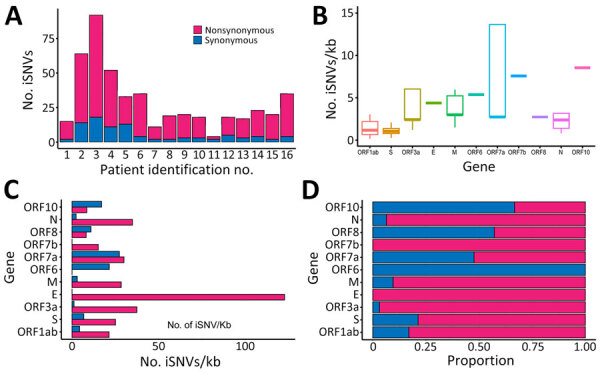
Distribution of iSNVs among patients in study of rapid emergence and evolution of SARS-CoV-2 intrahost variants among COVID-19 patients with prolonged infections, Singapore. A) Total number of iSNV detected in longitudinal samples from each patient, categorized as nonsynonymous or synonymous intrahost variants. B) Distribution plots of all iSNVs per kilobase among genes. Horizontal bars within boxes indicate medians; box tops and bottoms indicate upper and lower quartiles; vertical bars indicate minimum and maximum values. C) Overall iSNV counts across different genes with 5%­–95% frequency from longitudinal samples of all patients. D) Overall proportions of iSNVs among genes. E, envelope; iSNV, intrahost single-nucleotide variant; kb, kilobase; M, membrane; N, nucleocapsid; ORF, open reading frame; S, spike.

The 20 enrolled patients ranged in age from 21 to 70 (median 38 + 15.4) years, and body mass index ranged from 14.7 to 31.8 (median 25.8 + 5.0) kg/m^2^ (Tables 1, 2; Appendix 2 Figure 1). Hospital stays varied from 3 to 40 (median 7 + 10.2) days. Five patients (P2, P3, P5, P17, and P20) received remdesivir treatment. Four patients (P3, P4, P7, and P20) had underlying conditions, including hypertension, and experienced SARS-CoV-2 infections lasting 16 to 40 days (Table 1).

**Table 2:** Clinical features of patients in a study of rapid emergence and evolution of SARS-CoV-2 intrahost variants among COVID-19 patients with prolonged infections, Singapore*

Characteristics	All patients, n = 20
Median age, y (range)	38 (21–70)
Sex	
F	6 (30)
M	14 (70)
Healthcare worker	2 (10)
Median height, cm (range)	168 (151–185)
Median weight, kg (range)	69.2 (42.5–95.1)
Median BMI (range)	25.8 (14.7–31.8)
Hypertension	4 (20)
Intensive care unit admission	2 (10)
Median length of hospitalization, d (range)	7 (4–40)
Median CRP, mg/L (IQR)	41.53 (14.1–109.7)
Median leukocyte count, cells × 10^9^/L (range)	4.99 (2.5–21.7)
Median lymphocyte count, cells × 10^9^/L (IQR)	1.27 (0.97–1.68)
Remdesivir treatment	5 (25)
Long-term medication	4 (20)
International travel	8 (40)

*Values are no. (%) except as indicated. BMI, body mass index; CRP, C-reactive protein; IQR, interquartile range.

### iSNVs in Longitudinal SARS-CoV-2 Samples

We analyzed subconsensus de novo iSNVs in longitudinal samples from 16 COVID-19 patients. Of 198 sequenced samples, only 92 samples had sequencing depths of 200–62,000 reads, which we included for intrahost analysis. We excluded samples from 4 patients because reads were <200 or had inadequate coverage. Among the 16 included patients, we detected 4–108 iSNVs per patient at frequencies of 5%–95% (Appendix 1 Table 2) and more nonsynonymous than synonymous mutations (Figure 1, panel A). Two patients (P2, hospitalized for 30 days, and P3, hospitalized for 40 days) exhibited higher (>70) variant counts than other patients (Table 1; Figure 1, panel A).

Unique iSNVs were unevenly distributed across the genome. ORF7b and ORF10 exhibited moderately higher iSNVs per kilobase (Figure 1, panel B), and ORF1ab harbored the highest (n = 360) number of iSNVs compared with other gene regions (n = 4–60) (Appendix 1 Table 3). Within ORF1ab, nonsynonymous (n = 261) mutations exceeded synonymous (n = 61) mutations (Appendix 1 Table 4). Nonsynonymous mutations represented >50% of all variants in most genes, except for ORF6, ORF8, and ORF10 (Figure 1, panels C, D, Appendix 1 Table 4).

### Temporal Intrahost Dynamics of SARS-CoV-2 across Patients

**Figure 2:**
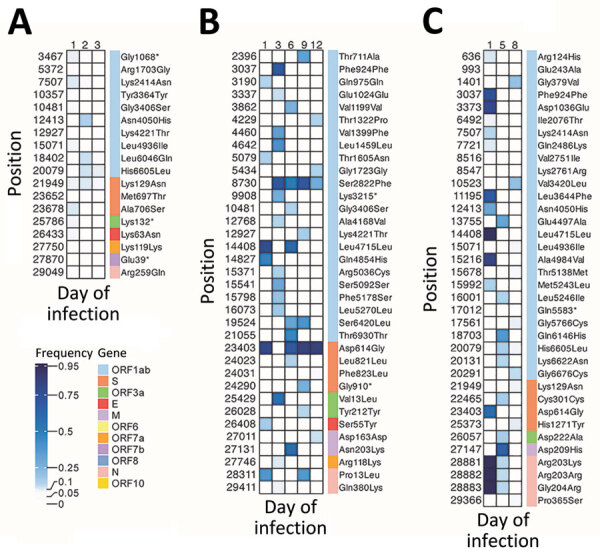
Variant heatmaps from individual patients in study of rapid emergence and evolution of SARS-CoV-2 intrahost variants among COVID-19 patients with prolonged infections, Singapore. A) Patient 1, infected with B6.6 lineage; B) patient 5, infected with B1.1 lineage; C) patient 16, infected with B1.1 lineage. Heatmaps show the frequency distribution of intrahost variants (5%–95%) identified in SARS-CoV-2 genomes from longitudinal samples collected in hospitalized patients during March–May 2020. Maps show corresponding genomic positions, associated genes, and amino acid changes. E, envelope; M, membrane; N, nucleocapsid; ORF, open reading frame; S, spike.

To assess the prevalence and distribution of de novo variants across SARS-CoV-2 genomes, we combined iSNV data from all longitudinal samples of 16 patients (Appendix 1 Table 1). Frequency plots revealed numerous minor variants at both low (5%–10%) and mid (10%–50%) frequencies and a notable decrease in iSNV count at >50% frequency (Appendix 2 Figure 2). We detected 9 high-frequency (>70%) variants, none of which were shared between patients. Conversely, we observed shared iSNVs in more than half the patients, and >11 shared variants detected at frequencies of 40%–70% (Appendix 2 Figure 2, panels A, B). For lower-frequency (5%–10%) variants, most were unique to individual patients, but a few were shared among multiple patients, including A7507C (ORF1a: K2414N), G10481A (ORF1a: G3406S), T15071A (ORF1b: L535I), T17190C (ORF1b: V1241A), T18402A (ORF1b: L1645Q), A20079T (ORF1b: H2204L), A21949C (spike: K129N), T23652C (spike: M697T), and A26433C (envelope: K63N) (Appendix 2 Figure 2, panel C). The K129N residues were in the N-terminal domain and the M697T residues were in the S2 subunit of the spike protein.

**Figure 3:**
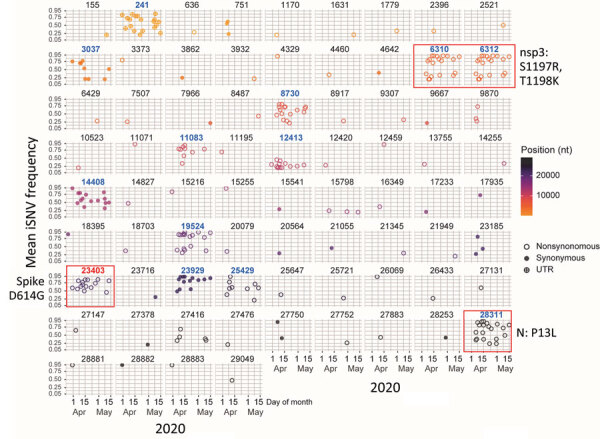
Temporal dynamics of 76 high-frequency iSNVs in study of rapid emergence and evolution of SARS-CoV-2 intrahost variants among COVID-19 patients with prolonged infections, Singapore. Colored closed circles represent synonymous variants; colored open circles represent nonsynonymous variants; crossed dots indicate variants at UTRs. Nucleotide positions of each iSNV are shown above the gray panels. The gradient of colored circles corresponds to iSNVs at respective nucleotide positions. Intrahost variants associated with persistent infections are highlighted in blue bold font, including the D614G intrahost variant (nucleotide position 23403), which marked is in red font above the corresponding open circles. Red rectangles indicate selected variants and their corresponding amino acid substitutions. iSNV, intrahost single-nucleotide variant; N, nucleocapsid; nsp, nonstructural protein; UTR, untranslated region.

**Figure 4:**
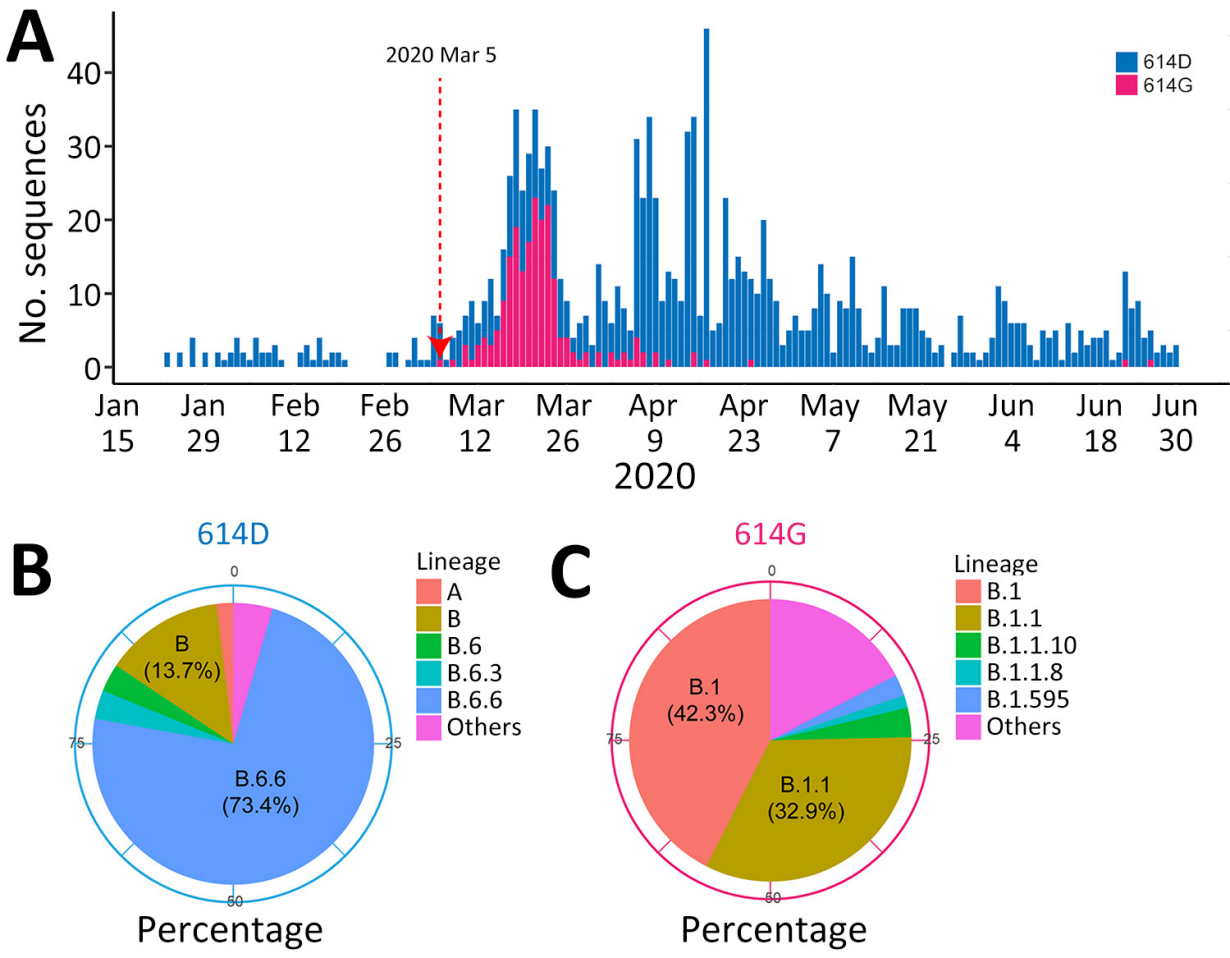
Evolutionary landscape in study of rapid emergence and evolution of SARS-CoV-2 intrahost variants among COVID-19 patients with prolonged infections, Singapore. A) Number of SARS-CoV-2 cases carrying the spike 614D or 614G mutations in all available SARS-CoV-2–positive samples. Dotted red line shows the first detection of the 614G mutation in Singapore. B, C) Percentages of different SARS-CoV-2 Pango lineages containing the 614D (B) or 614G (C) residues in the spike protein.

**Figure 5:**
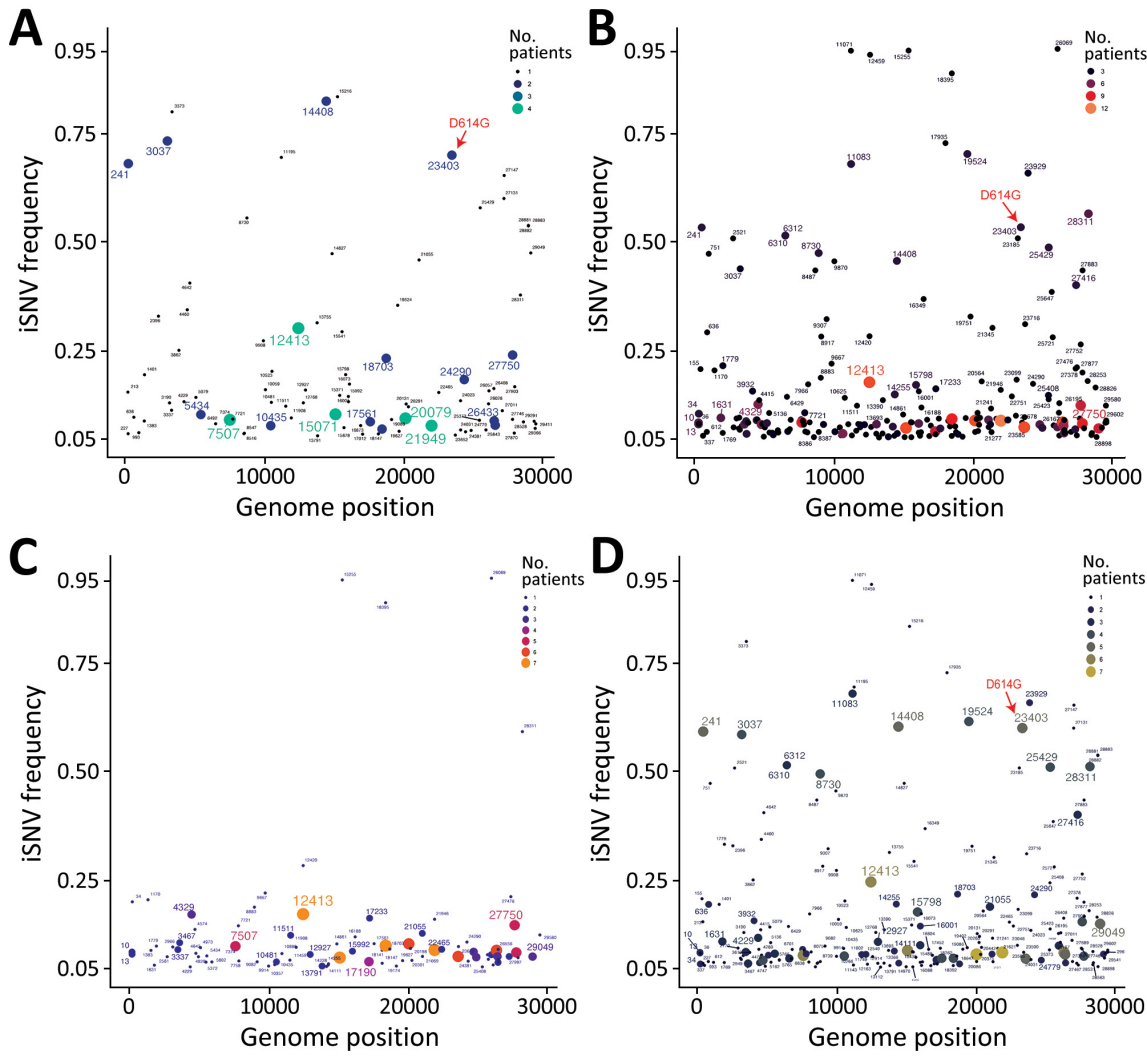
Comparative analysis of variants among lineages and infection durations in study of rapid emergence and evolution of SARS-CoV-2 iSNVs among COVID-19 patients with prolonged infections, Singapore. A, B) Variant frequency between B.1 lineages (A) and B.6 or B.6.6 lineages (B). Red arrows indicate the appearance of intrahost D614G spike variant at nucleotide position 23403. C, D) Variant frequency between COVID-19 patients with shorter infections (<7 days) (C) and those with prolonged infections (8–40 days) (D). Colored circles represent the number of patients with co-occurring intrahost variants; circle size is proportional to patient count. iSNVs, intrahost single-nucleotide variants.

**Figure 6:**
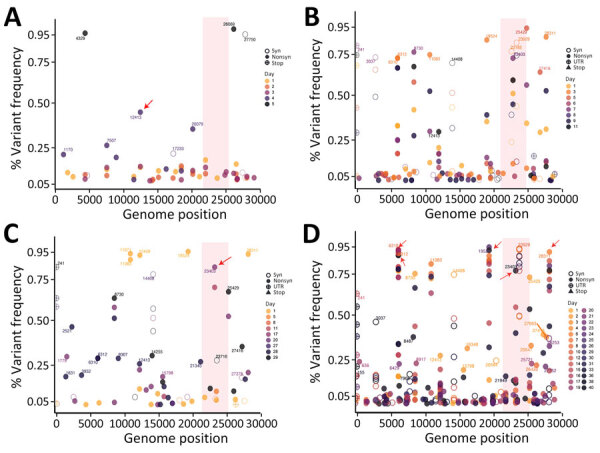
Temporal evolution of iSNVs in study of rapid emergence and evolution of SARS-CoV-2 intrahost variants among COVID-19 patients with prolonged infections, Singapore. The dot plots illustrate iSNVs detected over time and their fluctuations throughout the course of infection in 4 patients: A) patient 6, a 28-year-old man, who had a short infection and hospitalization lasting 7 days; B) patient 2, a 48-year-old man, who had a longer infection and hospitalization of 13 days; C) patient 4, a 65-year-old-man, who had a prolonged infection and hospitalization lasting 30 days; and D) patient 3, a 70-year-old man, who had a prolonged infection and hospitalization lasting 40 days. Colored gradient circles represent days of infections, and the shaded vertical bar indicates the spike region. Red arrows indicate nonsynonymous iSNVs that persisted at high frequency. iSNVs, intrahost single-nucleotide variants; nonsyn, nonsynonymous variants; syn, synonymous variants; UTR, untranslated region.

We observed a diverse array of iSNVs and substantial interpatient variability in both number and frequency (Figure 2; Appendix 2 Figures 3–6). Several patients, including P1, P8, P9, P13, P14, and P15, primarily harbored low-frequency (5%–20%) variants (Figure 2; Appendix 1 Table 1; Appendix 2 Figure 3). P1 exhibited more variants on day 1, most of which disappeared by day 2. That patient also harbored a unique spike substitution, A706S (Appendix 2 Figure 3), within the S2 subunit and had a short hospital stay of 5 days. By comparison, P5, who was older (>60 years of age) and hospitalized for 14 days, displayed a higher number of variants, particularly in the ORF1ab region, which appeared sporadically throughout infection (Figure 2; Appendix 2 Figure 3). That patient also carried a unique spike substitution at F823L. Patients with hospital stays >7 days, such as P2, P3, P4, P5, and P16, acquired more low-frequency variants (Figure 2; Appendix 2 Figures 3–6). Of note, P4 harbored a unique spike mutation at A397S within the receptor-binding domain of the spike protein as late as day 29 (Appendix 2 Figure 6), and P16 acquired a mutation, H1271Y, on day 8. In most patients, although some variants persisted, most either disappeared or appeared intermittently during infection.

During April–May 2020, we identified 76 variants with frequencies >20% in >1 sample (Figure 3). Because all patients were isolated, most variants likely emerged independently at specific time points. However, only 13 variants persisted during the early pandemic phase (Figure 3). Those variants included dual mutations at C6310A (nonstructural protein [NSP] 3: S1197R) and C6312A (NSP3: T1198K); co-occurrence in NSP3 has been associated with increased infection severity (*34*). Other persistent nonsynonymous variants included C8730T (NSP4: S59F), G11083T (NSP6: L37F), A12413C (NSP8: N108H), C19524T (NSP14: S495L), A23403G (spike: D614G), G25429T (ORF3a: V13L), and C28311T (N: P13L), suggesting those mutations were independently fixed. Among those mutations, the prominent spike D614G variant at nucleotide position 23403 might have emerged in multiple patients and coincided with S1197R (position 6310) and T1198K (position 6312), indicating a potential fitness advantage. The P13L mutation (position 28311) in the N gene has also been linked to reduced ICU admission and lower risk for death (*36*). Together, those findings highlight the emergence of diverse de novo synonymous and nonsynonymous variants in COVID-19 patients during the early phase of the pandemic.

To assess the local prevalence of the spike D614G mutation, we analyzed all available SARS-CoV-2 genomes from Singapore in 2020. The G variant of S614 was detected on March 5, 2020, and its prevalence increased substantially by mid-March (Figure 4, panel A). The 614G mutation was detected in several sublineages, predominantly in B.1 (42.3%) and B.1.1 (32.9%), and the 614D variant was predominant (73.4%) in the B.6.6 lineage (Figure 4, panels B, C; Appendix 1 Table 5).

### Differential Landscape of Intrahost Evolution between SARS-CoV-2 B.1 and B.6 Lineages

To investigate differences in intrahost evolution, we compared iSNV distributions in patients infected with B.1 or B.6/B.6.6 lineage viruses. The B.1 lineage exhibited fewer minor variants (iSNVs = 71) at 5%–20% frequency (Figure 5, panel A), whereas B.6/B.6.6 showed a marked increase (iSNVs = 185) (Figure 5, panel B). B.1 lineage also had fewer mid- to high-frequency (>20%) variants (n = 31) compared with B.6 (n = 60), although each lineage displayed a diverse set of shared high-frequency iSNVs.

In the B.1 lineage, several variants were shared among patients, including those at nucleotide positions 3037 (NSP3: F106F), 5434 (NSP3: G905G), 7507 (NSP3: K1596N), 14408 (NSP12: L323L), 15071 (NSP12: L544I), 18703 (NSP14: Q222H), 23403 (S: D614G), 20079 (NSP15: H153L), 21949 (spike: K129N), and 27750 (ORF7a: K119K) (Figure 5, panel A). In contrast, B.6/B.6.6 exhibited more low- to high-frequency iSNVs (Figure 5, panel B). However, we found only a few unique high-frequency (>20%) variants in 5 patients infected with B.6/B.6.6, including mutations at 6310 (NSP3: S1197R), 6312 (NSP3: T1198K), 11083 (NSP6: L37F), 19524 (NSP14: S495L), and 28311 (N: P13L). Spike D614G was observed at lower frequencies in B.6 patients compared with B.1.1 patients. Of note, 3 patients (P2, P3, and P4) acquired the S:D614G mutation during acute or postacute infection: P2 on day 1, P3 on day 3, and P4 as late as day 18 (Appendix 2 Figures 4–6). That time to acquisition suggests high-frequency variants might emerge over the course of infection, as in P3 and P4, who had B.6.6 lineage (Appendix 2 Figures 5, 6), but other variants might appear early, as in P16, who had B.1.1 lineage (Figure 2; Appendix 2 Figure 3).

### Prolonged SARS-CoV-2 Infection and Increasing Intrahost Genetic Variability

**Figure 7:**
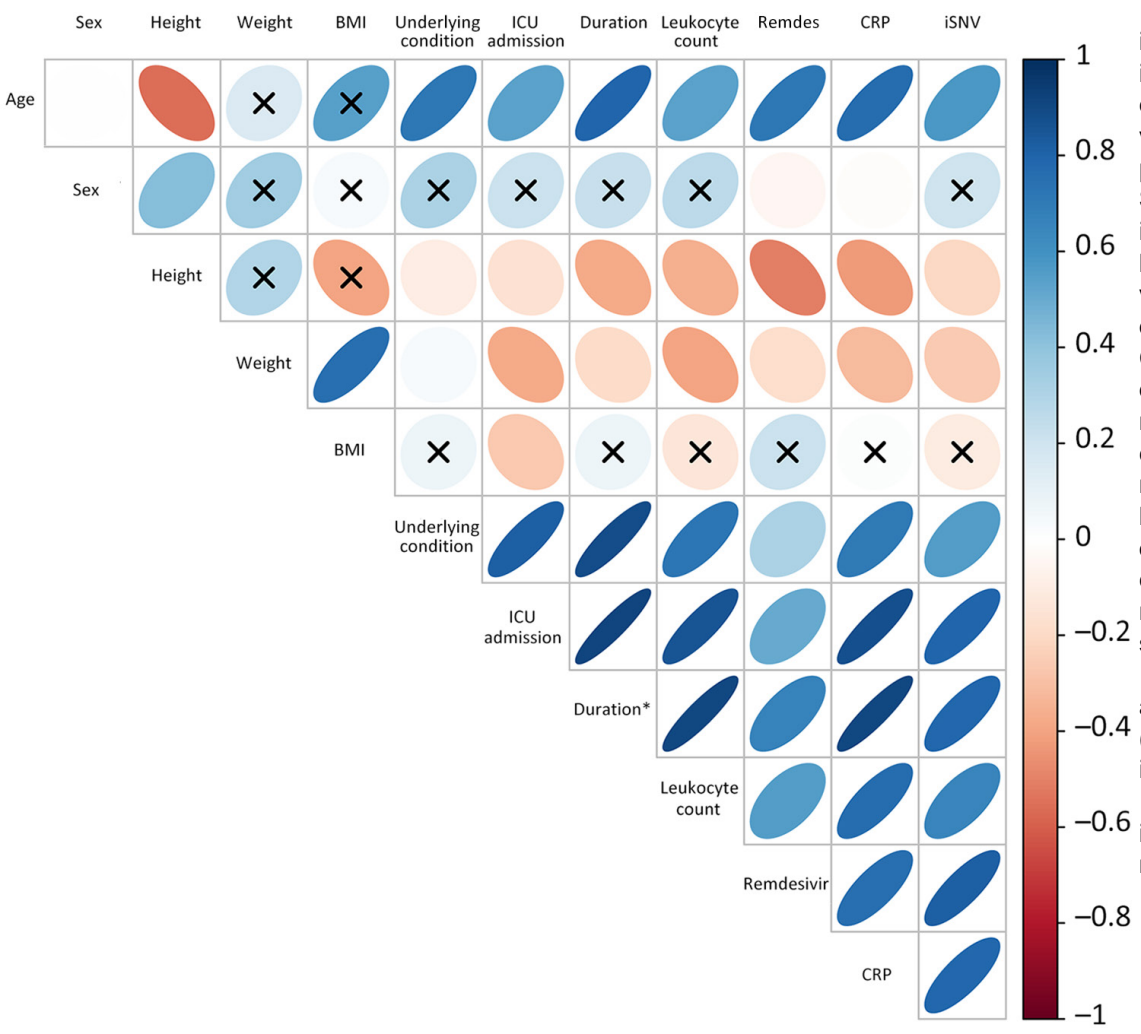
Correlation between iSNVs and clinical parameters in study of rapid emergence and evolution of SARS-CoV-2 intrahost variants among COVID-19 patients with prolonged infections, Singapore. Correlation matrix illustrates the relationships between the number of iSNVs and various clinical variables. Asterisk denotes duration of infection. Colored gradient indicates the degree of pairwise correlation with respect to Pearson correlation coefficient. Blue ellipsoids represent positive associations between any pairwise variables; orange ellipsoids denote negative correlations; the darker and narrower blue ellipsoids indicate stronger positive correlations. Black X denotes correlations that are not statistically significant (p>0.05). BMI, body mass index; CRP, C-reactive protein; ICU, intensive care unit; iSNVs, intrahost-single nucleotide variants; remdes, remdesivir.

We next compared de novo iSNVs in patients with infections <7 days versus those with 8–40 days of active infection. Patients with prolonged infections yielded more (n = 223) iSNVs across the genome than those with shorter infections (n = 93 iSNVs) (Figure 5, panels C, D). That difference was more pronounced in variants with >20% frequency (69 vs. 15). Among patients with shorter infections, most variants were at low (5%–20%) frequencies, and certain sites, such as 4329 (NSP3: I537T), 7507 (NSP3: K1596N), 17190 (NSP13: V318A), and 27750 (ORF7a: K119K), occurred sporadically. In contrast, prolonged infections exhibited 69 high-frequency (20%–80%) variants, although the fluctuation among those variants should be interpreted with caution. Notable nonsynonymous substitutions included D614G (S), S1197R and T1198K (NSP3), L37F (NSP6), V13L (ORF3a), and P13L (nucleocapsid [N]). To explore intrahost diversity during prolonged (>8 days) infection, we analyzed iSNVs during acute (<7 days) and nonacute phases. Many (n = 133) iSNVs emerged within 7 days, and most persisted beyond day 8 of infection (Appendix 2 Figure 7). Of note, patients with prolonged infections exhibited more iSNVs during the first week than those with shorter illness durations (Figure 5, panel C; Appendix 2 Figure 7).

We further examined intrahost SARS-CoV-2 evolution in individual patients. Most patients had numerous low-frequency iSNVs on day 1 (Figure 6; Appendix 2 Figures 8–10). We observed distinct patterns across patients: P6 (7-day hospitalization) showed low-frequency variants on days 2 and 3 and had few nonsynonymous variants (e.g., at nt position 12413) that were >25% by day 5 (Figure 6, panel A). P2 (13-day hospitalization) exhibited more iSNVs, many of which disappeared by day 8 (Figure 6, panel B). Both patients were infected with B.6.6, but P2 was older (48 years of age) and treated with remdesivir and P6 (28 years of age) was not treated (Table 1).

Two patients experienced prolonged infections; P4 had a 30-day infection, and P3 had a 40-day infection. P4 displayed several high-frequency nonsynonymous variants at positions 11071 and 11083 as early as day 1 (Figure 6, panel C), suggesting founder variants were present. In contrast, P3 showed many low-frequency iSNVs throughout infection, and only a few persisted beyond 3 weeks (Figure 6, panel D). Both patients were infected with lineage B.6.6. Specifically, in P3, the spike D614G variant fluctuated in frequency (Figure 6, panel D). It first appeared at 7% on day 3 (April 10, 2020), remained <18.2% for over a week, and then rose to 60.4% by day 15 (April 22, 2020) (Appendix 2 Figure 4). In contrast, patients with shorter (<7 days) infections (P1 and P7–P15) exhibited fewer iSNVs and limited frequency variation (Appendix 2 Figures 9, 10). Those findings highlight the variability in intrahost variant abundance and dynamics among patients.

### Correlation between iSNV Counts and Clinical Variables

Finally, we assessed Pearson correlations between iSNV counts and 11 clinical variables. We observed strong positive correlations with underlying conditions (*r* = 0.55), ICU admission (*r* = 0.80), infection duration (*r* = 0.78), remdesivir treatment (*r* = 0.81), leukocyte count (*r* = 0.66), and CRP (*r* = 0.78) (Table 3; Figure 7). Those variables also demonstrated strong intercorrelations, suggesting collinearity. Regression analysis further confirmed a statistically significant association between iSNV count and infection duration (p = 0.004) (Appendix 1 Table 6; Appendix 2 Figure 11). We observed no statistically significant differences between B.1 and B.6 lineages when comparing patient age or iSNV counts (Appendix 2 Figure 12). Collectively, those findings suggest host factors and treatment interventions influence the emergence of intrahost variants and contribute to viral genomic diversity.

**Table 3:** Pearson correlation matrix of iSNV counts and clinical characteristics patients in a study of rapid emergence and evolution of SARS-CoV-2 intrahost variants among COVID-19 patients with prolonged infections, Singapore*

Characteristic	Age	Sex	Height	Weight	BMI	Underlying conditions†	ICU admission	Infection duration	Leukocyte count	Remdesivir treatment	CRP	iSNV counts
Age	–	0.00	**–0.56**	0.16	0.54	**0.71**	**0.54**	**0.79**	**0.54**	**0.72**	**0.77**	**0.58**
Sex		–	**0.43**	0.35	0.04	0.32	0.22	0.22	0.26	**–0.05**	**–0.02**	0.21
Height			–	0.30	−0.39	**–0.09**	**–0.16**	**–0.38**	**–0.36**	**–0.50**	**–0.43**	**–0.21**
Weight				–	**0.75**	**0.04**	–**0.38**	**–0.18**	**–0.40**	**–0.18**	**–0.32**	**–0.26**
BMI					–	0.08	–**0.26**	0.08	−0.13	0.22	0.02	−0.10
Underlying conditions†						–	**0.81**	**0.88**	**0.73**	**0.32**	**0.70**	**0.55**
ICU admission							–	**0.92**	**0.85**	**0.51**	**0.88**	**0.80**
Infection duration								–	**0.90**	**0.66**	**0.91**	**0.78**
Leukocyte count									–	**0.55**	**0.76**	**0.66**
Remdesivir treatment										–	**0.76**	**0.81**
CRP											–	**0.78**
iSNV counts												–

*Bold text indicates p<0.05. BMI, body mass index; CRP, C-reactive protein; ICU, intensive care unit; iSNV, intrahost single nucleotide variant. †Including hypertension or hyperlipidemia.

## Discussion

As with most RNA viruses, SARS-CoV-2 undergoes rapid mutations and continuously generates de novo genetic variants, seeding sequential epidemics worldwide. In this study, we uncovered longitudinal intrahost dynamics of SARS-CoV-2 among hospitalized patients during the early months of the pandemic. Genomic analysis revealed a substantial number of intrahost variants emerged at varying frequencies from the first day of virus detection onwards. The low-frequency variants likely resulted from relaxed selection of a virus transmitting in an immunologically naive population or might be indicative of adaptation to the new human host. Relaxed selection on a virus population was previously observed in the first year of pandemic influenza A(H1N1) virus circulation in 2009, before the virus was subjected to immune-driven selection either from widespread infection or vaccination (*37*).

Intrahost population bottlenecks and natural selection play crucial roles in eliminating nonadvantageous variants (*24*). Several studies have indicated that intrahost variants show evidence of positive selection within persons who have persistent infections or chronic diseases or who are immunocompromised (*13*,*38*–*41*). Therefore, persistent infections might serve as suitable reservoirs for harboring de novo variants that can spread into the broader community. We showed that prolonged infections played a role in contributing to the broader range of genomic diversity within hosts. We also observed differential patterns of intrahost dynamics among Pango lineages. Of note, the presence of spike D614G in 3 patients with B.6 and B.6.6 lineages suggest that mutation evolved independently. However, because of stringent quarantine controls, those COVID-19 patients remained hospitalized until they tested negative by qPCR for 2 consecutive days before being discharged, preventing further transmission of that variant.

We also demonstrated that the magnitude of intrahost diversity was positively correlated with host and clinical factors. Higher leukocyte counts and increased CRP levels also have been associated with COVID-19 severity (*42*,*43*). Persistent SARS-CoV-2 infections have been shown to lead to extended periods of ongoing replication, enabling the virus to remain infectious and evolve immune escape mechanisms within hosts (*44*). In addition, older populations, particularly persons >65 years of age, might have impaired immune response, which has also been shown to result in a higher risk for long COVID (*45*) and an increased risk for reinfection with Omicron variants (*46*). Antiviral treatment has been suggested to contribute to greater levels of viral intrahost diversity (*47*).

The ongoing evolution and transmission of SARS-CoV-2 have triggered periodic epidemic waves in many countries, driven by the sequential emergence of variants over time and geographic space. Intrahost investigations have captured the dynamic patterns of population shifts, both longitudinally and cross-sectionally. Here, we showed the role of single-nucleotide variants in contributing to the overall genetic diversity and adaptive evolution of SARS-CoV-2 lineages. Collectively, both viral and host factors play major roles in the emergence and persistence of variants, which can increase the virus’s ability to evade immune-driven and vaccine-driven antibodies, displacing older lineages and potentially seeding future outbreaks. 

In conclusion, we identified shared SARS-CoV-2 variants across multiple patients and found that only a limited subset of high-frequency variants predominated and persisted throughout the course of infections. We also found that prolonged infections are positively associated with increased genetic diversity, underscoring the significant role of virus–host interactions in shaping intrahost variation and evolution. Enhanced genomic sequencing and monitoring should be prioritized for vulnerable populations, such older adults, immunocompromised persons, and persons living with chronic diseases. The data generated from this study provide crucial insights into the emergence and transmission of de novo variants and can inform the development of effective vaccine candidates and strategies for protection.

Appendix 1 Additional parameters from study of rapid emergence and evolution of SARS-CoV-2 intrahost variants among COVID-19 patients with prolonged infections, Singapore

Appendix 2 Additional information on rapid emergence and evolution of SARS-CoV-2 intrahost variants among COVID-19 patients with prolonged infections, Singapore.

## References

[1] Su YCF, Anderson DE, Young BE, Linster M, Zhu F, Jayakumar J, et al. Discovery and genomic characterization of a 382-nucleotide deletion in ORF7b and ORF8 during the early evolution of SARS-CoV-2. MBio. 2020;11:e01610–20. 10.1128/mBio.01610-20 32694143 PMC7374062

[2] Lin RJ, Lee TH, Lye DC. From SARS to COVID-19: the Singapore journey. Med J Aust. 2020;212:497–502.e1. 10.5694/mja2.50623 32474940 PMC7300591

[3] Mazur-Panasiuk N, Rabalski L, Gromowski T, Nowicki G, Kowalski M, Wydmanski W, et al. Expansion of a SARS-CoV-2 Delta variant with an 872 nt deletion encompassing *ORF7a*, *ORF7b* and *ORF8*, Poland, July to August 2021. Euro Surveill. 2021;26:22. 10.2807/1560-7917.ES.2021.26.39.2100902 34596017 PMC8485581

[4] Tang Z, Yu P, Guo Q, Chen M, Lei Y, Zhou L, et al. Clinical characteristics and host immunity responses of SARS-CoV-2 Omicron variant BA.2 with deletion of ORF7a, ORF7b and ORF8. Virol J. 2023;20:106. 10.1186/s12985-023-02066-3 37248496 PMC10226014

[5] Feng Y, Zhao X, Luo T, Chen Z, Yang H, Chen N, et al. Emergence of a SARS-CoV-2 Omicron subvariant BA.2.2 with a 454-nucleotide genomic deletion—Sichuan Province, China, May 10, 2022. China CDC Wkly. 2022;4:904–6. 10.46234/ccdcw2022.098 36285323 PMC9579981

[6] Niemeyer D, Stenzel S, Veith T, Schroeder S, Friedmann K, Weege F, et al. SARS-CoV-2 variant Alpha has a spike-dependent replication advantage over the ancestral B.1 strain in human cells with low ACE2 expression. PLoS Biol. 2022;20:e3001871. 10.1371/journal.pbio.3001871 36383605 PMC9710838

[7] Ke R, Martinez PP, Smith RL, Gibson LL, Mirza A, Conte M, et al. Daily longitudinal sampling of SARS-CoV-2 infection reveals substantial heterogeneity in infectiousness. Nat Microbiol. 2022;7:640–52. 10.1038/s41564-022-01105-z 35484231 PMC9084242

[8] Lythgoe KA, Hall M, Ferretti L, de Cesare M, MacIntyre-Cockett G, Trebes A, et al.; Oxford Virus Sequencing Analysis Group (OVSG); COVID-19 Genomics UK (COG-UK) Consortium. SARS-CoV-2 within-host diversity and transmission. Science. 2021;372:eabg0821. 10.1126/science.abg0821 33688063 PMC8128293

[9] Valesano AL, Rumfelt KE, Dimcheff DE, Blair CN, Fitzsimmons WJ, Petrie JG, et al. Temporal dynamics of SARS-CoV-2 mutation accumulation within and across infected hosts. PLoS Pathog. 2021;17:e1009499. 10.1371/journal.ppat.1009499 33826681 PMC8055005

[10] Tonkin-Hill G, Martincorena I, Amato R, Lawson ARJ, Gerstung M, Johnston I, et al.; COVID-19 Genomics UK (COG-UK) Consortium; Wellcome Sanger Institute COVID-19 Surveillance Team. Patterns of within-host genetic diversity in SARS-CoV-2. eLife. 2021;10:e66857. 10.7554/eLife.66857 34387545 PMC8363274

[11] Weigang S, Fuchs J, Zimmer G, Schnepf D, Kern L, Beer J, et al. Within-host evolution of SARS-CoV-2 in an immunosuppressed COVID-19 patient as a source of immune escape variants. Nat Commun. 2021;12:6405. 10.1038/s41467-021-26602-3 34737266 PMC8568958

[12] Khateeb D, Gabrieli T, Sofer B, Hattar A, Cordela S, Chaouat A, et al. SARS-CoV-2 variants with reduced infectivity and varied sensitivity to the BNT162b2 vaccine are developed during the course of infection. PLoS Pathog. 2022;18:e1010242. 10.1371/journal.ppat.1010242 35020754 PMC8789181

[13] Li J, Du P, Yang L, Zhang J, Song C, Chen D, et al. Two-step fitness selection for intra-host variations in SARS-CoV-2. Cell Rep. 2022;38:110205. 10.1016/j.celrep.2021.110205 34982968 PMC8674508

[14] Kemp SA, Collier DA, Datir RP, Ferreira IATM, Gayed S, Jahun A, et al.; CITIID-NIHR BioResource COVID-19 Collaboration; COVID-19 Genomics UK (COG-UK) Consortium. SARS-CoV-2 evolution during treatment of chronic infection. Nature. 2021;592:277–82. 10.1038/s41586-021-03291-y 33545711 PMC7610568

[15] Voloch CM, da Silva Francisco R Jr, de Almeida LGP, Brustolini OJ, Cardoso CC, Gerber AL, et al. Intra-host evolution during SARS-CoV-2 prolonged infection. Virus Evol. 2021;7:veab078. 10.1093/ve/veab078 34642605 PMC8500031

[16] Corman VM, Landt O, Kaiser M, Molenkamp R, Meijer A, Chu DKW, et al. Detection of 2019 novel coronavirus (2019-nCoV) by real-time RT-PCR. Euro Surveill. 2020;25:2000045. 10.2807/1560-7917.ES.2020.25.3.2000045 31992387 PMC6988269

[17] Bolger AM, Lohse M, Usadel B. Trimmomatic: a flexible trimmer for Illumina sequence data. Bioinformatics. 2014;30:2114–20. 10.1093/bioinformatics/btu170 24695404 PMC4103590

[18] Li H, Durbin R. Fast and accurate short read alignment with Burrows-Wheeler transform. Bioinformatics. 2009;25:1754–60. 10.1093/bioinformatics/btp324 19451168 PMC2705234

[19] Okonechnikov K, Golosova O, Fursov M; UGENE team. Unipro UGENE: a unified bioinformatics toolkit. Bioinformatics. 2012;28:1166–7. 10.1093/bioinformatics/bts091 22368248

[20] O’Toole Á, Scher E, Underwood A, Jackson B, Hill V, McCrone JT, et al. Assignment of epidemiological lineages in an emerging pandemic using the pangolin tool. Virus Evol. 2021;7:veab064. 10.1093/ve/veab064 34527285 PMC8344591

[21] Li H, Handsaker B, Wysoker A, Fennell T, Ruan J, Homer N, et al.; 1000 Genome Project Data Processing Subgroup. The Sequence Alignment/Map format and SAMtools. Bioinformatics. 2009;25:2078–9. 10.1093/bioinformatics/btp352 19505943 PMC2723002

[22] Koboldt DC, Zhang Q, Larson DE, Shen D, McLellan MD, Lin L, et al. VarScan 2: somatic mutation and copy number alteration discovery in cancer by exome sequencing. Genome Res. 2012;22:568–76. 10.1101/gr.129684.111 22300766 PMC3290792

[23] Raglow Z, Surie D, Chappell JD, Zhu Y, Martin ET, Kwon JH, et al.; Investigating Respiratory Viruses in the Acutely Ill (IVY) Network. SARS-CoV-2 shedding and evolution in patients who were immunocompromised during the omicron period: a multicentre, prospective analysis. Lancet Microbe. 2024;5:e235–46. 10.1016/S2666-5247(23)00336-1 38286131 PMC11849777

[24] Wang Y, Wang D, Zhang L, Sun W, Zhang Z, Chen W, et al. Intra-host variation and evolutionary dynamics of SARS-CoV-2 populations in COVID-19 patients. Genome Med. 2021;13:30. 10.1186/s13073-021-00847-5 33618765 PMC7898256

[25] Schirmer M, D’Amore R, Ijaz UZ, Hall N, Quince C. Illumina error profiles: resolving fine-scale variation in metagenomic sequencing data. BMC Bioinformatics. 2016;17:125. 10.1186/s12859-016-0976-y 26968756 PMC4787001

[26] Cingolani P, Platts A, Wang L, Coon M, Nguyen T, Wang L, et al. A program for annotating and predicting the effects of single nucleotide polymorphisms, SnpEff: SNPs in the genome of *Drosophila melanogaster strain w*^1118^; iso-2; iso-3. Fly (Austin). 2012;6:80–92. 10.4161/fly.19695 22728672 PMC3679285

[27] Gu H, Quadeer AA, Krishnan P, Ng DYM, Chang LDJ, Liu GYZ, et al. Within-host genetic diversity of SARS-CoV-2 lineages in unvaccinated and vaccinated individuals. Nat Commun. 2023;14:1793. 10.1038/s41467-023-37468-y 37002233 PMC10063955

[28] Gonzalez-Reiche AS, Alshammary H, Schaefer S, Patel G, Polanco J, Carreño JM, et al.; PARIS/PSP study group. Sequential intrahost evolution and onward transmission of SARS-CoV-2 variants. Nat Commun. 2023;14:3235. 10.1038/s41467-023-38867-x 37270625 PMC10239218

[29] Markov PV, Ghafari M, Beer M, Lythgoe K, Simmonds P, Stilianakis NI, et al. The evolution of SARS-CoV-2. Nat Rev Microbiol. 2023;21:361–79. 10.1038/s41579-023-00878-2 37020110

[30] Gu Z, Gu L, Eils R, Schlesner M, Brors B. *circlize* Implements and enhances circular visualization in R. Bioinformatics. 2014;30:2811–2. 10.1093/bioinformatics/btu393 24930139

[31] Wölfel R, Corman VM, Guggemos W, Seilmaier M, Zange S, Müller MA, et al. Virological assessment of hospitalized patients with COVID-2019. Nature. 2020;581:465–9. 10.1038/s41586-020-2196-x 32235945

[32] Young BE, Ong SWX, Kalimuddin S, Low JG, Tan SY, Loh J, et al.; Singapore 2019 Novel Coronavirus Outbreak Research Team. Epidemiologic features and clinical course of patients infected with SARS-CoV-2 in Singapore. JAMA. 2020;323:1488–94. 10.1001/jama.2020.3204 32125362 PMC7054855

[33] Hu B, Guo H, Zhou P, Shi ZL. Characteristics of SARS-CoV-2 and COVID-19. Nat Rev Microbiol. 2021;19:141–54. 10.1038/s41579-020-00459-7 33024307 PMC7537588

[34] Lamers MM, Haagmans BL. SARS-CoV-2 pathogenesis. Nat Rev Microbiol. 2022;20:270–84. 10.1038/s41579-022-00713-0 35354968

[35] Venables WNRB. Modern applied statistics with S. 4th ed. New York: Springer; 2002.

[36] Alsuwairi FA, Alsaleh AN, Alsanea MS, Al-Qahtani AA, Obeid D, Almaghrabi RS, et al. Association of SARS-CoV-2 nucleocapsid protein mutations with patient demographic and clinical characteristics during the Delta and Omicron waves. Microorganisms. 2023;11:1288. 10.3390/microorganisms11051288 37317262 PMC10224071

[37] Su YCF, Bahl J, Joseph U, Butt KM, Peck HA, Koay ESC, et al. Phylodynamics of H1N1/2009 influenza reveals the transition from host adaptation to immune-driven selection. Nat Commun. 2015;6:7952. 10.1038/ncomms8952 26245473 PMC4918339

[38] Ghafari M, Hall M, Golubchik T, Ayoubkhani D, House T, MacIntyre-Cockett G, et al.; Wellcome Sanger Institute COVID-19 Surveillance Team; COVID-19 Infection Survey Group; COVID-19 Genomics UK (COG-UK) Consortium. Prevalence of persistent SARS-CoV-2 in a large community surveillance study. Nature. 2024;626:1094–101. 10.1038/s41586-024-07029-4 38383783 PMC10901734

[39] Choi B, Choudhary MC, Regan J, Sparks JA, Padera RF, Qiu X, et al. Persistence and evolution of SARS-CoV-2 in an immunocompromised host. N Engl J Med. 2020;383:2291–3. 10.1056/NEJMc2031364 33176080 PMC7673303

[40] Chaguza C, Hahn AM, Petrone ME, Zhou S, Ferguson D, Breban MI, et al.; Yale SARS-CoV-2 Genomic Surveillance Initiative. Accelerated SARS-CoV-2 intrahost evolution leading to distinct genotypes during chronic infection. Cell Rep Med. 2023;4:100943. 10.1016/j.xcrm.2023.100943 36791724 PMC9906997

[41] Wagner C, Kistler KE, Perchetti GA, Baker N, Frisbie LA, Torres LM, et al. Positive selection underlies repeated knockout of ORF8 in SARS-CoV-2 evolution. Nat Commun. 2024;15:3207. 10.1038/s41467-024-47599-5 38615031 PMC11016114

[42] Wang G, Wu C, Zhang Q, Wu F, Yu B, Lv J, et al. C-reactive protein level may predict the risk of COVID-19 aggravation. Open Forum Infect Dis. 2020;7:ofaa153. 10.1093/ofid/ofaa153 32455147 PMC7197542

[43] Bhargava A, Fukushima EA, Levine M, Zhao W, Tanveer F, Szpunar SM, et al. Predictors for severe COVID-19 infection. Clin Infect Dis. 2020;71:1962–8. 10.1093/cid/ciaa674 32472676 PMC7314166

[44] Hettle D, Hutchings S, Muir P, Moran E; COVID-19 Genomics UK (COG-UK) consortium. Persistent SARS-CoV-2 infection in immunocompromised patients facilitates rapid viral evolution: Retrospective cohort study and literature review. Clin Infect Pract. 2022;16:100210. 10.1016/j.clinpr.2022.100210 36405361 PMC9666269

[45] Mansell V, Hall Dykgraaf S, Kidd M, Goodyear-Smith F. Long COVID and older people. Lancet Healthy Longev. 2022;3:e849–54. 10.1016/S2666-7568(22)00245-8 36480981

[46] Breznik JA, Rahim A, Zhang A, Ang J, Stacey HD, Bhakta H, et al. Early Omicron infection is associated with increased reinfection risk in older adults in long-term care and retirement facilities. EClinicalMedicine. 2023;63:102148. 10.1016/j.eclinm.2023.102148 37753447 PMC10518514

[47] Heyer A, Günther T, Robitaille A, Lütgehetmann M, Addo MM, Jarczak D, et al. Remdesivir-induced emergence of SARS-CoV2 variants in patients with prolonged infection. Cell Rep Med. 2022;3:100735. 10.1016/j.xcrm.2022.100735 36075217 PMC9378267

