# Supplementary material for: Rapid Emergence and Evolution of SARS-CoV-2 Intrahost Variants among COVID-19 Patients with Prolonged Infections, Singapore
Source: Emerg Infect Dis. 2025 Aug;31(8):1537–49. doi: 10.3201/eid3108.241419 (PMC12309778; doi:10.3201/eid3108.241419)
Supplement: Appendix 2 — Additional information on rapid emergence and evolution of SARS-CoV-2 intrahost variants among COVID-19 patients with prolonged infections, Singapore. [file 24-1419-Techapp-s2.pdf]

Article DOI: https://doi.org/10.3201/eid3108.241419

EID cannot ensure accessibility for supplementary materials supplied by authors. Readers who have difficulty accessing supplementary content should contact the authors for assistance.

Rapid Emergence and Evolution of SARS-CoV-2 Intrahost Variants among COVID-19 Patients with Prolonged Infections, Singapore

Appendix 2

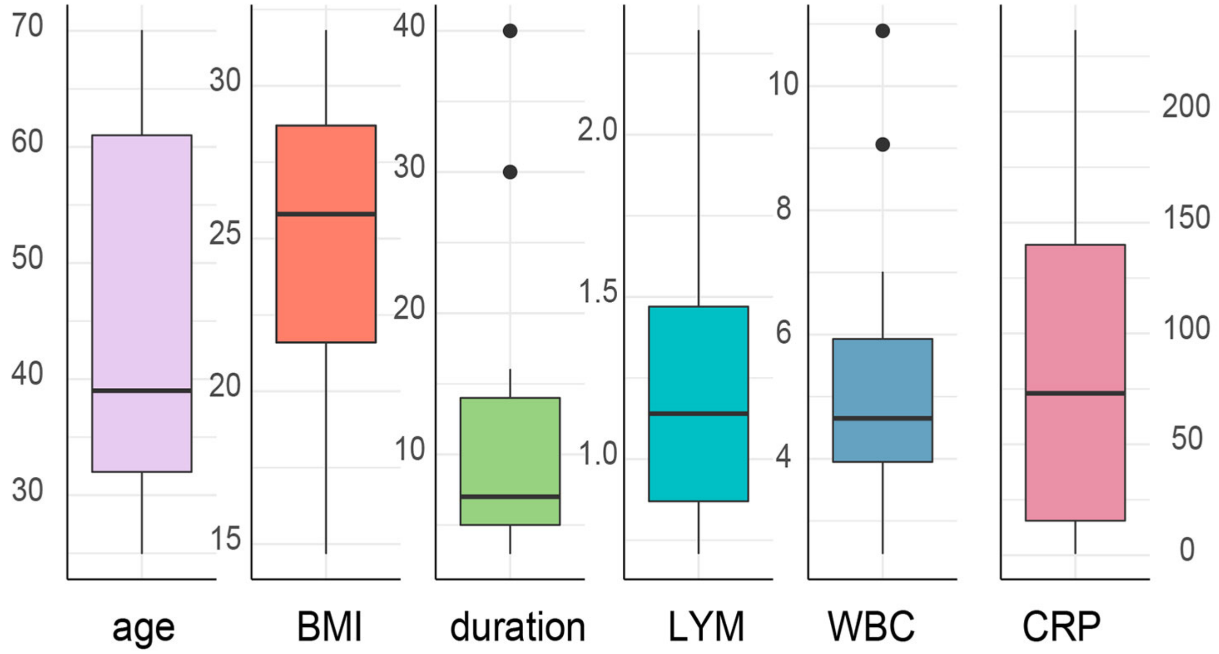

Appendix 2 Figure 1. Clinical characteristics of COVID-19 patients in Singapore. Box plots illustrate the distribution of patient age (years), body mass index (BMI), duration of infection (days), lymphocyte count (LYM, $\times 10^9$L), white blood cell count (WBC, $\times 10^9$/L), and C-reactive protein (CRP, mg/L).

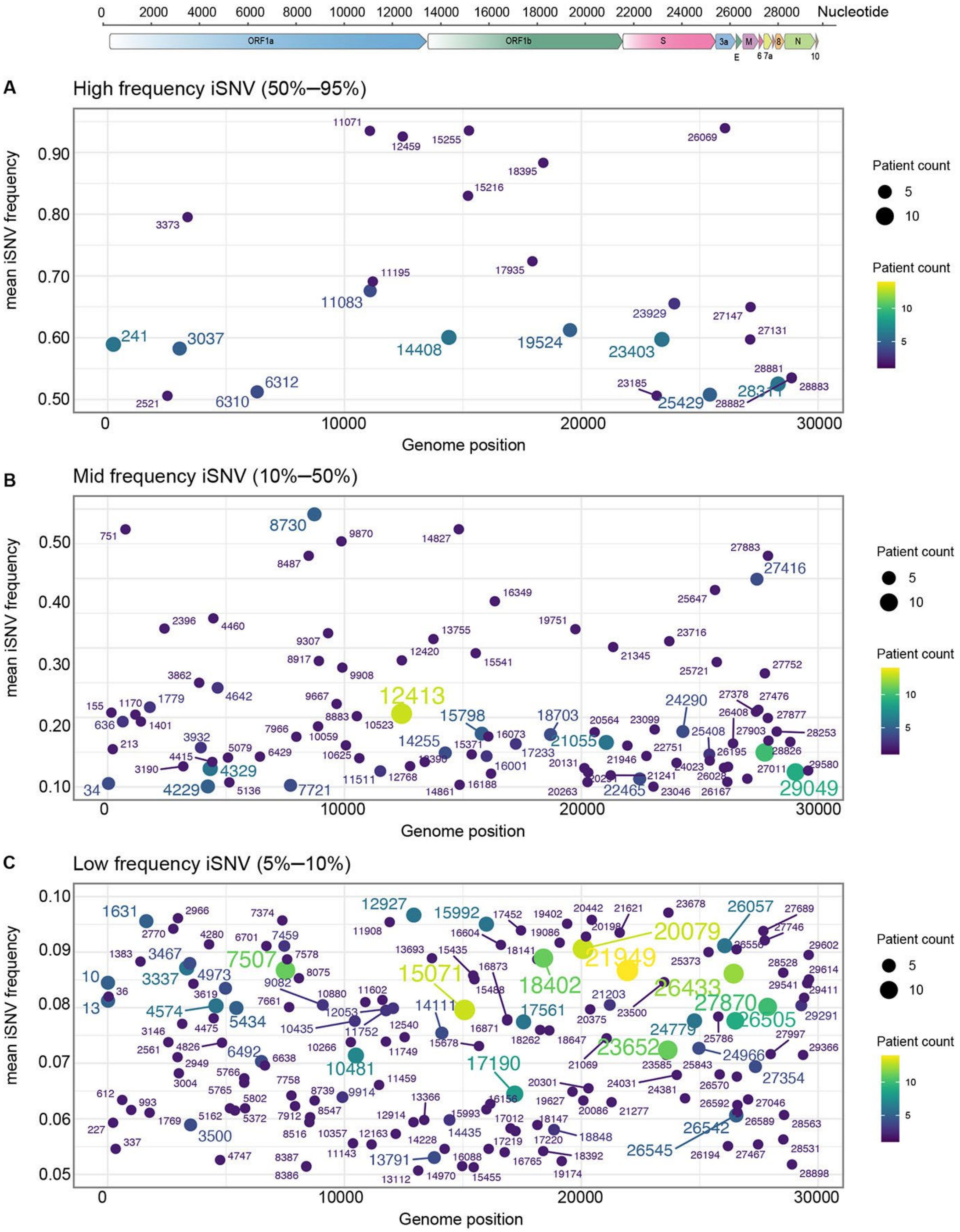

Appendix 2 Figure 2. Intrahost evolutionary landscape of SARS-CoV-2 virus. Distribution of intrahost variants identified in SARS-CoV-2 genomes from longitudinal samples among hospitalized patients collected between March and May 2020. Variants at different frequencies are shown including A) 50%–95%, B) 10%–50%, and C) 5%–10%. The colored circles and their sizes represent the number of patients with co-occurring intrahost variants. The number at each dot indicate the nucleotide position of intrahost variant within the genome.

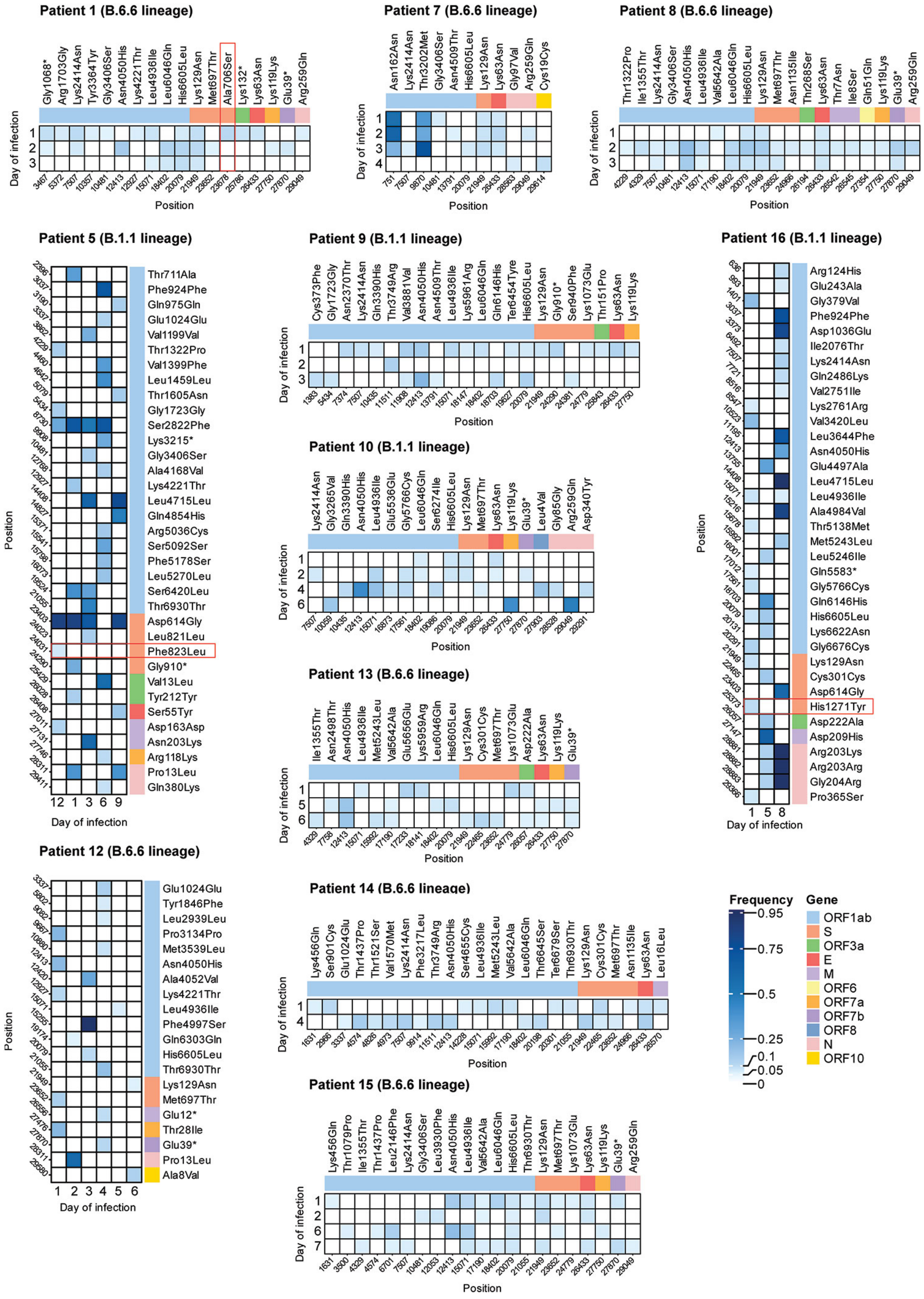

Appendix 2 Figure 3. Detection of SARS-CoV-2 intrahost variants in individual patients. Heatmaps show the frequency distribution of intrahost variants (5%–95%) identified in SARS-CoV-2 genomes from longitudinal samples collected in hospitalized patients between March and May 2020. Each panel represents a single patient. Columns correspond to genomic positions where variants were detected, with the colored bar indicating the associated gene. The corresponding amino acid changes are also specified (* indicates that the variant is synonymous). Rows represent sampling time points, measured in days post-baseline. A red rectangle highlights a unique spike variant identified in one patient.

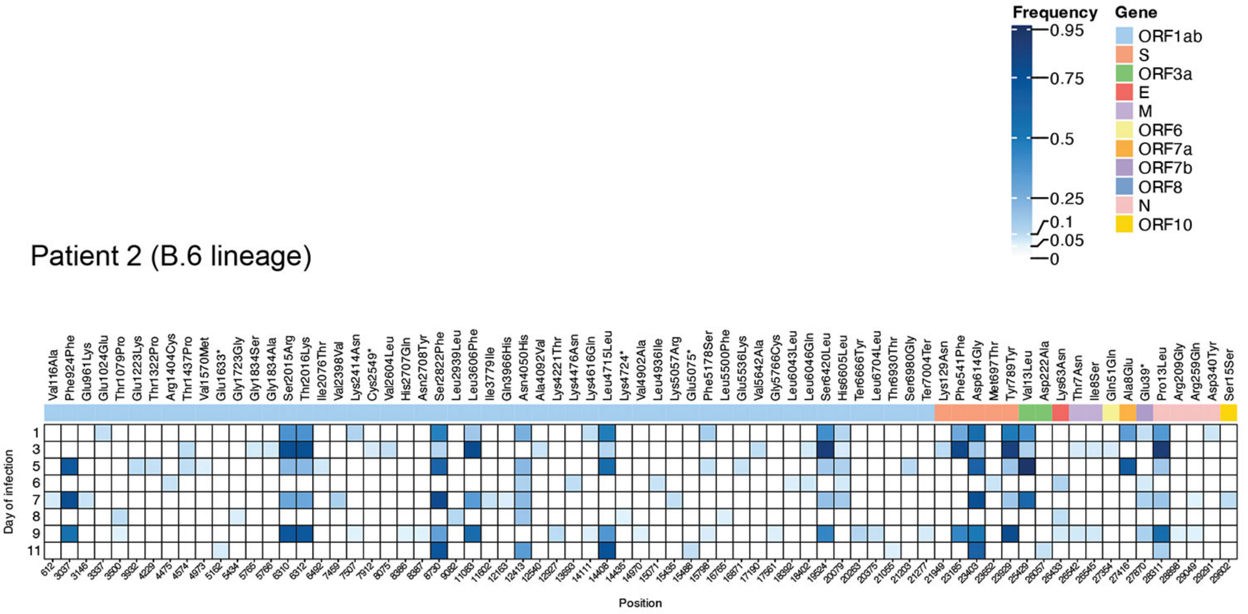

Appendix 2 Figure 4. Intrahost genomic diversity and evolution of SARS-CoV-2 virus in Patient 2. Frequency distribution of intrahost variants (5–95%) detected in SARS-CoV-2 genomes from longitudinal samples of Patient 2. Columns correspond to genomic positions where variants were detected, with the colored bar indicating the associated gene. The corresponding amino acid substitution at each position is specified (* indicates that the variant is synonymous). Rows represent sampling time points, measured as the number of days post-baseline.

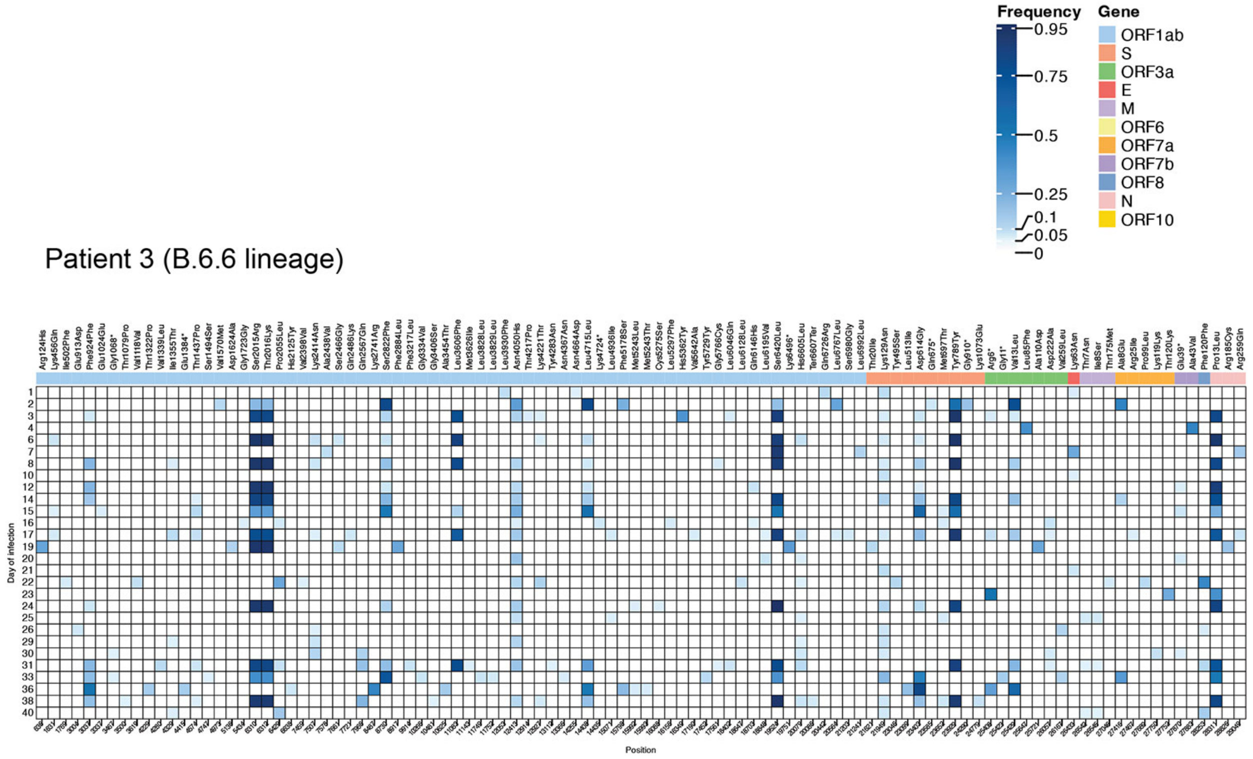

Appendix 2 Figure 5. Intrahost genomic diversity and evolution of SARS-CoV-2 virus in Patient 3. Frequency distribution of intrahost variants (5%–95%) detected in SARS-CoV-2 genomes from longitudinal samples of Patient 3. Columns correspond to genomic positions where variants were detected, with the colored bar indicating the associated gene. The corresponding amino acid substitution at each position is specified (* indicates that the variant is synonymous). Rows represent sampling time points, measured as the number of days post-baseline.

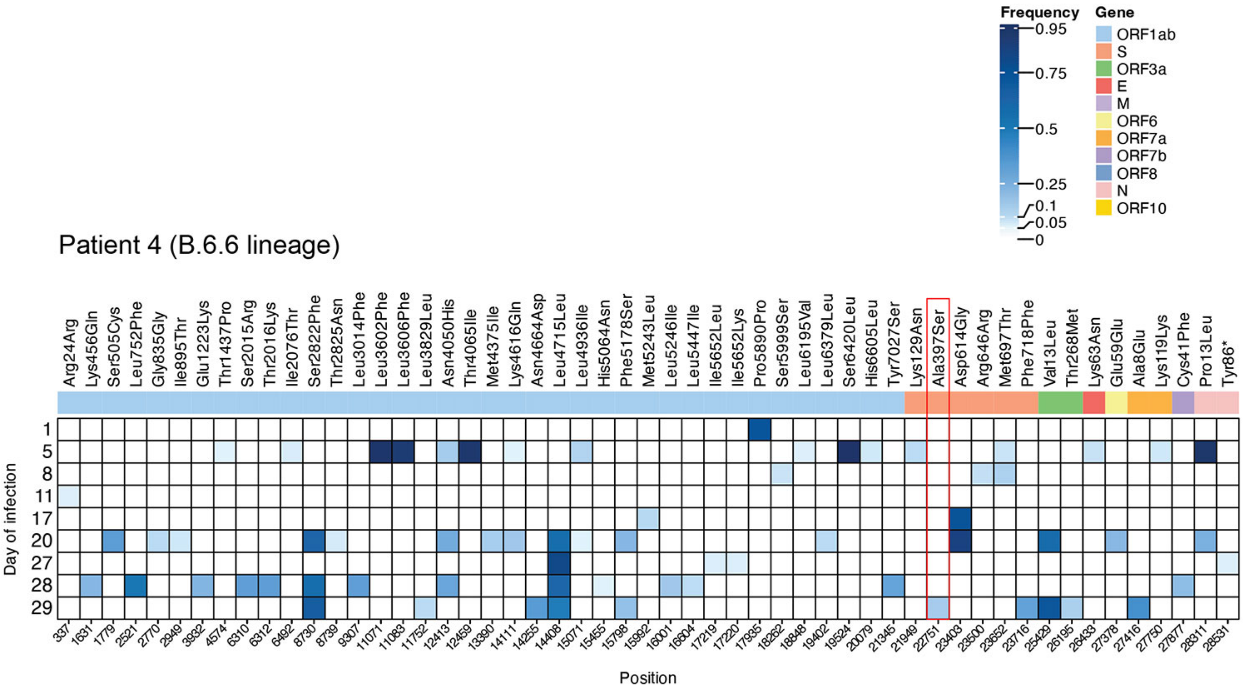

Appendix 2 Figure 6. Intrahost genomic diversity and evolution of SARS-CoV-2 virus in Patient 4. Frequency distribution of intrahost variants (5–95%) detected in SARS-CoV-2 genomes from longitudinal samples of Patient 4. Columns correspond to genomic positions where variants were detected, with the colored bar indicating the associated gene. The corresponding amino acid substitution at each position is specified (* indicates that the variant is synonymous). Rows represent sampling time points, measured as the number of days post-baseline. Red rectangle denotes a unique spike variant identified in a single patient.

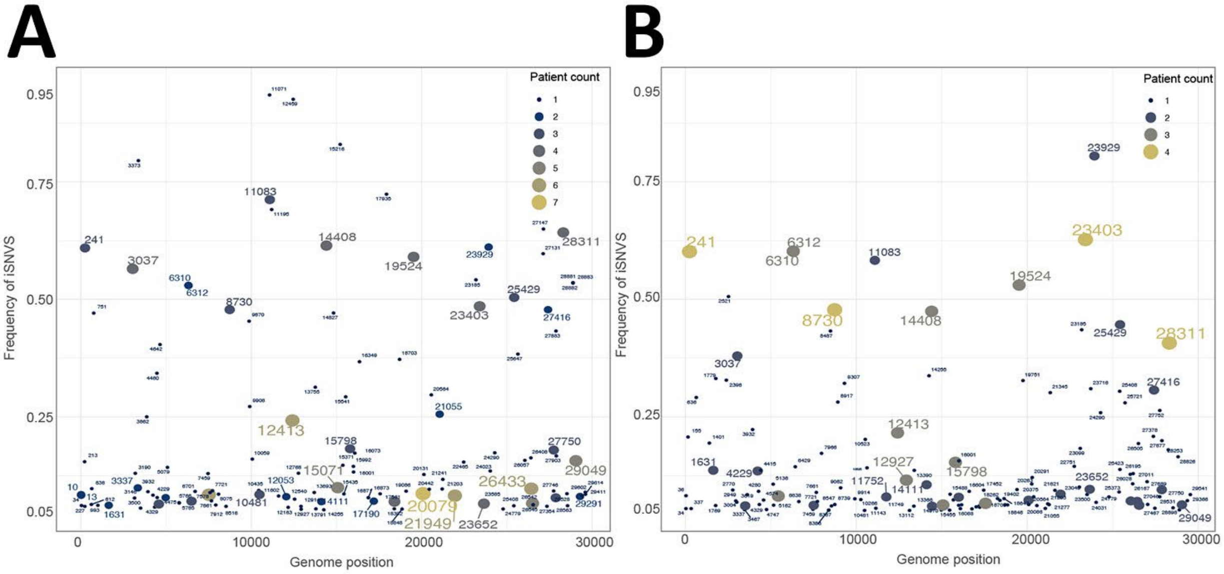

Appendix 2 Figure 7. Comparison of iSNV diversity in patients with prolonged infections. This figure compares iSNV diversity between A) the acute phase (first 7 days) and B) the non-acute phase (beyond 8 days). The colored circles and their sizes represent the number of patients with co-occurring intrahost variants. The number at each dot indicate the nucleotide position of intrahost variant within the genome.

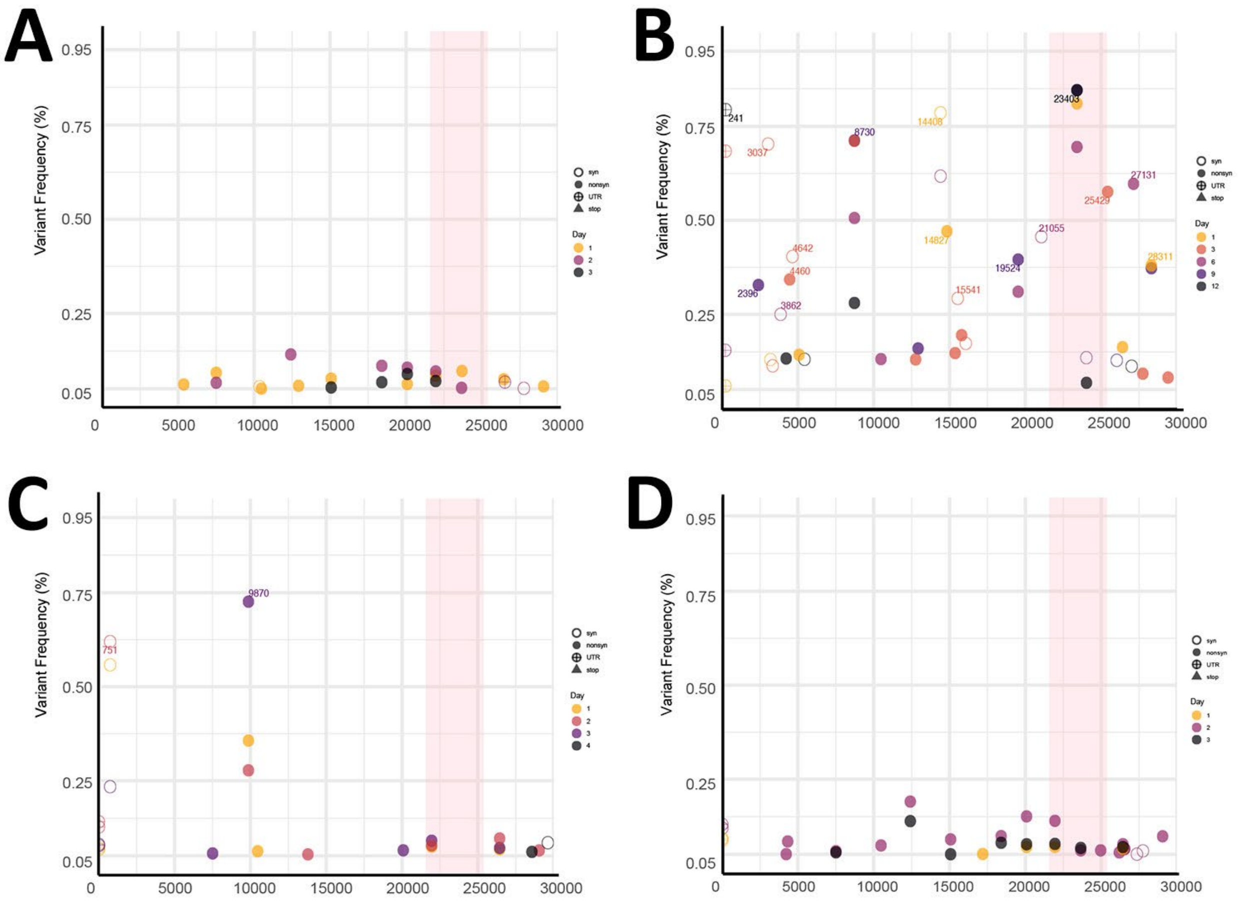

Appendix 2 Figure 8. Temporal evolution of intrahost variants in individual COVID-19 patients (P1, P5, P7 and P8) in Singapore. Abbreviations: syn, synonymous variants; non-syn, non-synonymous variants; stop, stop codon variants. Pink vertical bars represent spike region.

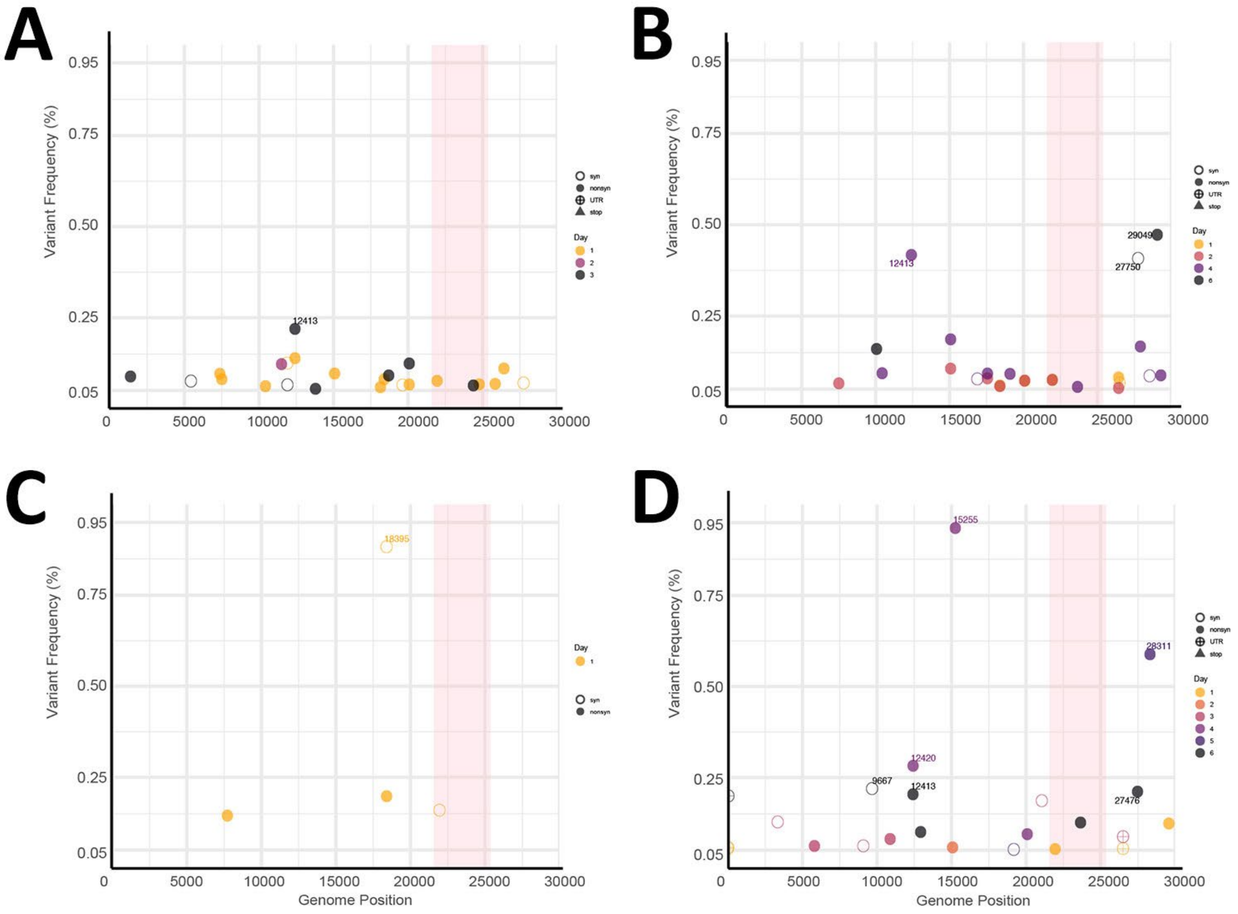

Appendix 2 Figure 9. Temporal evolution of intrahost variants in individual COVID-19 patients (P9, P10, P11 and P12) in Singapore. Abbreviations: syn, synonymous variants; non-syn, non-synonymous variants; stop, stop codon variants. Pink vertical bars represent spike region.

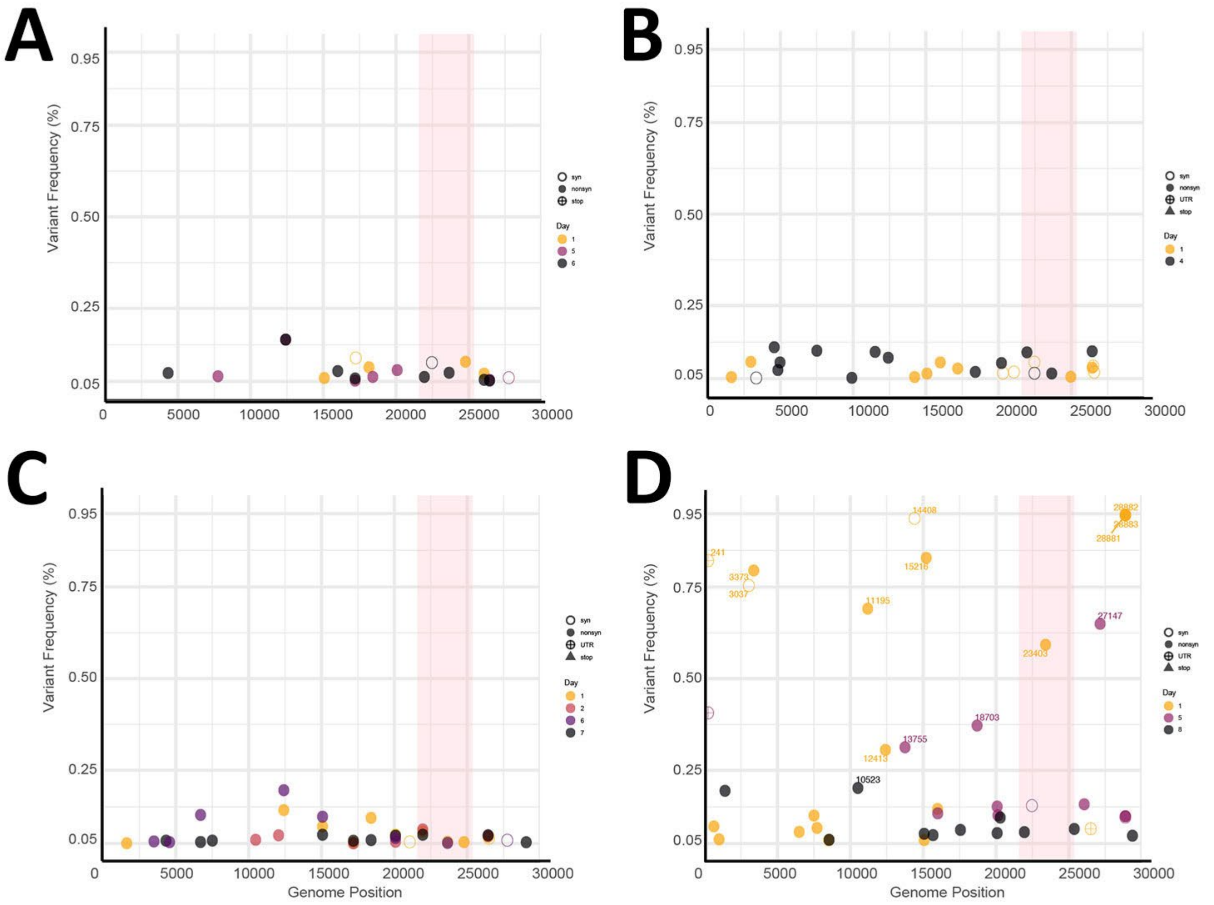

Appendix 2 Figure 10. Temporal evolution of intrahost variants in individual COVID-19 patients (P13, P14, P15 and P16) in Singapore. Abbreviations: syn, synonymous variants; non-syn, non-synonymous variants; stop, stop codon variants. Pink vertical bars represent spike region.

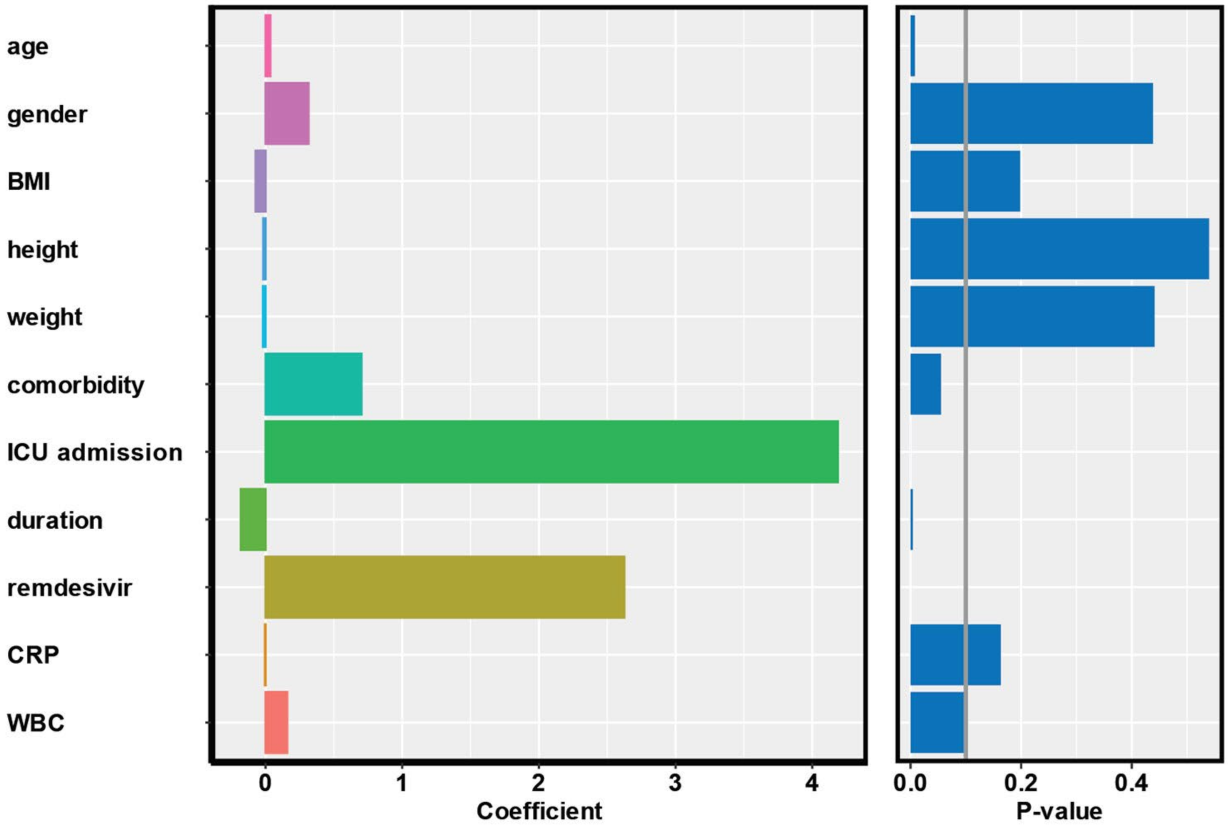

Appendix 2 Figure 11. Linear regression model fitted with generalized least squares (GLS) showing association between iSNV counts and clinical parameters of COVID-19 patients. Coefficients and support estimated using generalized linear squares (GLS) model. Grey vertical line indicates the P-value of <0.05.

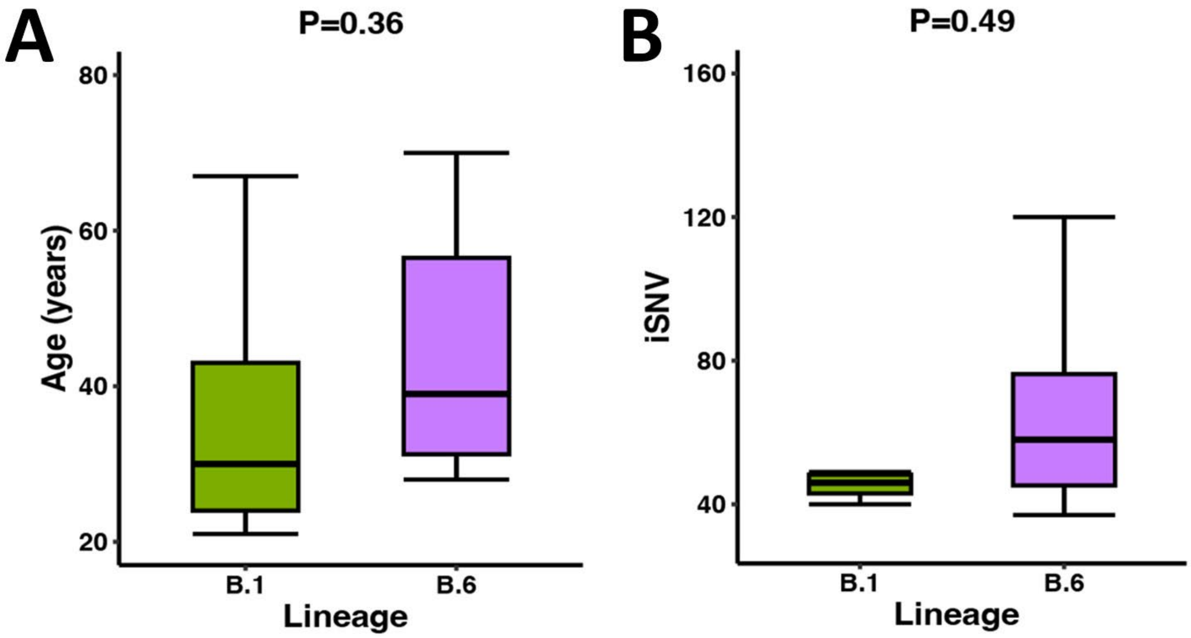

Appendix 2 Figure 12. Age and iSNV counts differ between infection duration and lineage. A) Differences in age (years) between Pango lineages. B) Differences in iSNV count between Pango lineages. Statistical significance was calculated using the Wilcoxon test and P values were false discovery fate (FDR) corrected using the Banjamini-Hochberg method.
